# Isolating the effect of confounding from the observed survival benefit of screening participants — a methodological approach illustrated by data from the German mammography screening programme

**DOI:** 10.1186/s12916-024-03258-6

**Published:** 2024-01-30

**Authors:** Laura Buschmann, Ina Wellmann, Nadine Bonberg, Jürgen Wellmann, Hans-Werner Hense, André Karch, Heike Minnerup

**Affiliations:** 1 Institute of Epidemiology and Social Medicine, University of Münster, Münster, Germany; 2 Cancer Registry of North Rhine-Westphalia, Bochum, Germany

**Keywords:** Breast cancer, Mammography screening, Survival, Bias, Confounding

## Abstract

**Background:**

Mammography screening programmes (MSP) aim to reduce breast cancer mortality by shifting diagnoses to earlier stages. However, it is difficult to evaluate the effectiveness of current MSP because analyses can only rely on observational data, comparing women who participate in screening with women who do not. These comparisons are subject to several biases: one of the most important is self-selection into the MSP, which introduces confounding and is difficult to control for. Here, we propose an approach to quantify confounding based on breast cancer survival analyses using readily available routine data sources.

**Methods:**

Using data from the Cancer Registry of North Rhine-Westphalia, Germany, we estimate the relative contribution of confounding to the observed survival benefit of participants of the German MSP. This is accomplished by comparing non-participants, participants with screen-detected and participants with interval breast cancers for the endpoints “death from breast cancer” and “death from all causes other than breast cancer” — the latter being assumed to be unrelated to any MSP effect. By using different contrasts, we eliminate the effects of stage shift, lead and length time bias. The association of breast cancer detection mode with survival is analysed using Cox models in 68,230 women, aged 50–69 years, with breast cancer diagnosed in 2006–2014 and followed up until 2018.

**Results:**

The hazard of dying from breast cancer was lower in participants with screen-detected cancer than in non-participants (HR = 0.21, 95% CI: 0.20–0.22), but biased by lead and length time bias, and confounding. When comparing participants with interval cancers and non-participants, the survival advantage was considerably smaller (HR = 0.62, 95% CI: 0.58–0.66), due to the elimination of stage shift and lead time bias. Finally, considering only mortality from causes other than breast cancer in the latter comparison, length time bias was minimised, but a survival advantage was still present (HR = 0.63, 95% CI: 0.56–0.70), which we attribute to confounding.

**Conclusions:**

This study shows that, in addition to stage shift, lead and length time bias, confounding is an essential component when comparing the survival of MSP participants and non-participants. We further show that the confounding effect can be quantified without explicit knowledge of potential confounders by using a negative control outcome.

**Supplementary Information:**

The online version contains supplementary material available at 10.1186/s12916-024-03258-6.

## Background

The aim of mammography screening programmes (MSPs) is to reduce breast cancer mortality by shifting diagnoses to an earlier, prognostically better tumour stage (“stage shift”) [1–3]. Evidence for the effectiveness of mammography screening comes from randomised controlled trials (RCTs) that have been carried out more than 20 years ago [3, 4]. The transferability of this evidence to the current situation might be limited, as in the meantime survival, particularly in higher tumour stages, has increased substantially because of more effective treatment options [5, 6]. On the other hand, the technique and thus sensitivity of mammography have improved [5, 7], leading to a slightly longer mean sojourn time and lower tumour stages at diagnosis [8]. As MSPs have now been implemented in many countries, including Germany, where women aged 50–69 years have been able to participate in the MSP every 2 years since its introduction in 2009, any new evidence regarding the effectiveness of MSPs must be generated from post-screening observational studies. All observational studies must handle the problem of self-selection, also called “healthy screenee bias” [9–11], which, using a more technical term, effectively is confounding. This confounding results from differences between women who do and who do not participate in screening, regarding their risk of developing breast cancer (difference in incidence) as well as dying from breast cancer (difference in survival). Most post hoc studies compare breast cancer mortality between screening participants and non-participants with little or no information on confounder variables and use so-called correction factors to adjust for confounding, which, however, rely mainly on historical data [10–12]. Another way to assess the effectiveness of ongoing MSPs is to use survival analyses to compare the survival of MSP participants and non-participants diagnosed with breast cancer [13–15]. In survival analyses, the problem of confounding has usually not been sufficiently addressed, with most studies focussing on lead and length time bias [13–17]. However, individual studies have also concluded that although 50–70% can be attributed to stage shift and the aforementioned biases, at least 30% remain unexplained [14]. Lead time bias reflects the spurious survival benefit of screen-detected tumours that is caused by the addition of a lead time to the actual survival time in screening participants. Lead time hereby refers to the period between the detection of an asymptomatic tumour in screening and the (virtual) point in time when the same tumour would have been diagnosed without an early detection programme because of symptoms. Length time bias, on the other hand, results from potential differences in tumour growth rates across the different detection modes. Slower growing and thus potentially less aggressive tumours are screen-detectable for a longer time than fast-growing and presumably more aggressive tumours, which leads to an overrepresentation of tumours with a better prognosis and thus longer survival in women with screen-detected breast cancer. The extent of lead and length time bias in the overall survival benefit of screening participants has been estimated in various studies, and correction procedures such as by Duffy et al. [15], Abrahamsson et al. [16] and Vratanar and Pohar Perme [17] have been applied to compensate for these two biases in survival analyses, but not for confounding bias.

In the present analysis, we aim to show, that the comparison of breast cancer-specific survival of MSP participants and non-participants is additionally biased by substantial confounding, i.e., self-selection bias, which results from potential group differences that manifest after the diagnosis of breast cancer and affect breast cancer survival, e.g., via therapy adherence or comorbidities [18, 19]. We further show that survival analyses based on readily available cancer registry data can be used to isolate and thereby quantify the extent of confounding in a population, without requiring specific information on confounders or control groups, but by using information on interval cancers and a negative control outcome, that is, death from all causes other than breast cancer.

We use data from the largest German cancer registry, the Cancer Registry of North Rhine-Westphalia (LKR NRW). The LKR NRW has a proven track record of providing high-quality cancer registry data in a source population of 18 million inhabitants. Based on these data, we isolate the effect of confounding from the observed overall survival benefit of MSP participants using information on interval breast cancers and deaths from all causes other than breast cancer.

## Methods

### Study population

Routine data from the LKR NRW, Germany, were used to extract information on all women aged 50–69 years in the state of North Rhine-Westphalia (NRW) with a diagnosis of breast cancer in the period from 2006 to 2014 and with a follow-up on causes of death until December 31st, 2018. Breast cancer was defined as an incident diagnosis of C50 according to the International Statistical Classification of Diseases and Related Health Problems, 10th revision (ICD-10) [20]. Metastases and relapses were not counted as incident breast cancer cases. The dataset comprised information on death (cause of death, date of death), tumour (time of first diagnosis, tumour stage according to TNM classification [21]), and detection mode (exposure).

### Exposure

We defined three mutually exclusive exposure categories based on detection mode: (1) Screen-detected breast cancer was defined as a breast cancer that was detected at screening. (2) Interval breast cancer was defined as breast cancer in screening participants that was not detected at screening, but after or between two regular screening rounds with a maximum of 30 months after the last screening date. The cut-off of 30 months was chosen to include women who wanted to attend regular screening but for various reasons did not manage to do so within exactly 24 months. (3) Breast cancer in non-participants comprised all incident breast cancers in women who did not participate in the MSP. Women who participated in MSP, but whose breast cancer was diagnosed more than 30 months after the last screening examination, were not included in our analyses.

### Outcome

In addition to “death from breast cancer (ICD-10: C50)”, the negative control outcome “death from all causes other than breast cancer (ICD-10: C50)” was also observed between 2006 until 2018. We further used the endpoint “death from causes other than breast cancer (ICD-10: C50) and cardiovascular diseases (ICD-10: I00-I99)” in sensitivity analyses, to account for potential misclassification of women who died because of breast cancer but whose certified cause of death was cardiovascular disease [22, 23].

### Study design

Before disentangling the different causes of an observed survival benefit of screened women, we first analysed descriptively the distribution of tumour stages in women with incident breast cancers after the introduction of the MSP in NRW, Germany. We then identified evidence of lead and length time as well as confounding using survival plots, before isolating and estimating the effect of confounding in the final step by comparing survival across women with different breast cancer detection modes and causes of death.

**Fig. 1:**
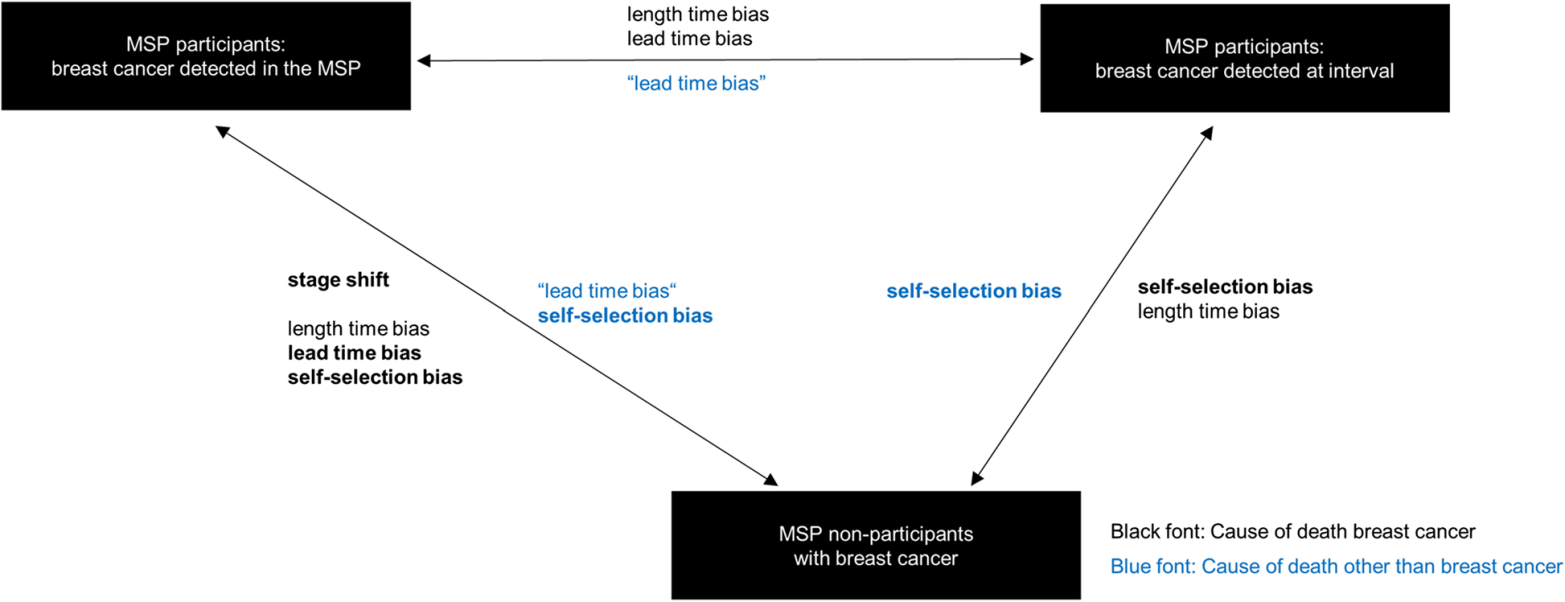
Hypothesis of this study: Self-selection bias can be isolated from other forms of bias by comparing participants with interval breast cancers with non-participants with breast cancer regarding death from all causes other than breast cancer. As shown by bold and normal print, the extent and even direction of the assumed lead and length time biases can differ across the different comparisons. Lead Time Bias: Lead time of unlimited length (maximum is the study period) can be expected in the comparison of survival across MSP participants with screen-detected tumours and non-participants. In the comparison of MSP participants with screen-detected breast cancer and MSP participants with interval breast cancers, the lead time can only reach a maximum of 2.5 years, equivalent to the time interval that defines an interval cancer. In the same two comparisons, a sort of “lead time” is also expected when considering death from all causes other than breast cancer, due to the advanced start of the observation period in MSP participants with screen-detected breast cancers. Length Time Bias: Length time bias might also be present in all two-way comparisons. However, the effect and even direction cannot be well predicted, as the tumours in patients with interval cancers most probably reflect a highly heterogeneous sample regarding growth rate and prognostic factors [24]. For death from all causes other than breast cancer, however, there is no length time bias. *MSP *Mammography screening programme

The hypothesis underlying the proposed isolation of the effect of self-selection is shown in Fig. 1. Women with breast cancer can be unequivocally assigned to one of three detection modes depending on MSP participation and the time when the tumour is diagnosed: (1) participants with tumours detected in the MSP, (2) participants with tumours detected in the interval between two regular screenings (up to 30 months after the last participation in the MSP), and (3) tumours detected in non-participants. Women with breast cancer can also be assigned to one of two mutually exclusive endpoints, i.e., death from breast cancer or death from all causes other than breast cancer. Else, they can survive the given observation period. Depending on the endpoint (“death from breast cancer” or the negative control outcome “death from all causes other than breast cancer”) the two-way comparisons of survival rates across the three detection modes are affected by different combinations of biases and causal (intended) effects, respectively. The intended effect of the MSP, i.e., the shift to earlier tumour stages should be observable in the comparison of breast cancer survival between women with tumours detected in the MSP and non-participants with breast cancer. This same comparison, however, is also affected by length time bias, lead time bias and self-selection bias (confounding). The consideration of interval cancer now offers two complementary comparisons to further separate the effect of the different biases. To isolate the effect of self-selection, the comparison of non-participants suffering from breast cancer with participants whose breast cancer was diagnosed in the interval must be considered. Compared to the comparison of participants with tumours detected in the MSP and participants with tumours detected in the interval, the comparison of participants with tumours detected in the interval and non-participants with breast cancer is free of any intended effects (stage shift). It is also free of lead time, as the tumours here are detected not as a consequence of a screening procedure, but because of symptoms. The remaining biases in the comparison of breast cancer survival between women with interval cancers and non-participating women with breast cancer are self-selection and length time bias. The direction of the latter, however, cannot easily be foreseen in patients with interval cancers, as the tumours probably present a widely heterogeneous group regarding growth rate and prognostic factors. Bordás et al. [24], for example, hypothesise that interval breast cancers comprise a sample of fast-growing, higher-grade tumours as well as a sample of benign slow-growing cancers with very long sojourn times that are missed at screening. These two groups would relate to different amounts and even directions of length time compared to non-participants. We suppose, therefore, that a rather small overall effect of length time bias can be expected to affect the comparison of women with interval tumours and non-participants. If we now consider alternative causes of death — instead of death from breast cancer — in this comparison, we can effectively minimise the length time bias. The only bias that might now distort the survival of MSP participants with interval cancers compared to non-participants is self-selection/confounding.

Our hypothesis comes with several assumptions, which we will address in more detail in the discussion. First, we assume that women with interval cancers are a random subgroup of all MSP participants regarding potential confounders. Second, we assume that interval cancers and breast cancers in non-participants are detected because of symptoms. Third, we assume that death from all causes other than breast cancer is affected by the same confounders or the same magnitude of confounding, respectively, than death from breast cancer.

### Statistical analyses

Column charts visualise the tumour stages across the years 2006–2014 stratified by detection mode. Differences in (stage-specific) survival rates according to participation status as well as detection mode were visualised with Aalen-Johansen plots. Cox models were fitted to analyse the association between detection mode and the outcomes “death from breast cancer” as well as “death from all causes other than breast cancer”. The models used different contrasts of participation status and detection mode as explanatory variables: (1) binary contrast of MSP participants (comprising women with screen-detected cancers as well as women with interval-detected cancers) versus MSP non-participants, (2) three-category contrast of MSP non-participants versus MSP participants with interval-detected cancers versus MSP participants with screen-detected cancers. Regarding adjustment: All Cox models were fitted in an unadjusted way, as well as adjusted for age at diagnosis and year of diagnosis. For the endpoint “death from breast cancer”, we also fitted models that were additionally adjusted for the tumour stage, to correct for stage shift. For the contrast “participants versus non-participants” we further fitted models that were stratified by tumour stage. In all survival analyses, time zero was defined as the day of breast cancer diagnosis. The time resolution (and therewith the time scale of the analyses) is given in days. Since the data from the LKR NRW are assumed to provide complete follow-up, individuals were censored on the day of death or after December 31, 2018, whichever came first. All analyses were carried out using SAS 9.4 TS Level 1M4.

### Sensitivity analyses

Since the official statistics on causes of death in Germany are only of average quality, misclassifications may occur [25, 26]. It is to be expected that some breast cancer deaths were misclassified as deaths due to cardiovascular diseases, the most common registered cause of death in Germany [27]. To test the robustness of our results regarding potential misclassification of the outcome, all Cox models were also calculated with the endpoint “Death from causes other than breast cancer (ICD-10: C50) and cardiovascular diseases (ICD-10: I00-I99)”.

## Results

A total of 68,230 women between the ages of 50 and 69 years were diagnosed with breast cancer in NRW in the period from 2006 to 2014. Of these cancer cases, 24,418 were screen-detected, 7795 were detected in the interval, and 36,017 were diagnosed in MSP non-participants (Table 1). Across all detection modes and years, breast cancer was most frequently detected in the T1 stage, and case numbers generally decreased with increasing tumour stage (Table 1, Additional file 1: Fig. S1).

**Table 1:** Characterisation of the study group of women (50–69 years) with an incident breast cancer diagnosis (C50) in the period 2006–2014 and a follow-up on causes of death until December 31st, 2018, with and without stratification by detection mode

Characteristics	Participants			Non- participants	Total
	**Screen-detected BC** ***N* = 24,418**	**Interval-detected BC** ***N* = 7795**	**Screen- and interval-detected BC** ***N* = 32,213**	***N* = 36,017**	***N* = 68,230**
**Age at diagnosis**					
Median, IQR	61 (10)	60 (9)	60 (10)	60 (10)	60 (19)
**Year of diagnosis, *N* (%)**^a^					
2006	640 (2.6)	22 (0.3)	662 (2.1)	5664 (15.7)	6326 (9.3)
2007	2017 (8.3)	184 (2.4)	2201 (6.8)	5963 (16.6)	8164 (12.0)
2008	3142 (12.9)	644 (8.3)	3786 (11.8)	5131 (14.2)	8917 (13.1)
2009	3135 (12.8)	937 (12.0)	4072 (12.6)	4163 (11.6)	8235 (12.1)
2010	3109 (12.7)	1174 (15.1)	4283 (13.3)	3421 (9.5)	7704 (11.3)
2011	2970 (12.2)	1168 (15.0)	4138 (12.8)	3262 (9.1)	7400 (10.8)
2012	3299 (13.5)	1187 (15.2)	4486 (13.9)	2910 (8.1)	7396 (10.8)
2013	3072 (12.6)	1263 (16.2)	4335 (13.5)	2891 (8.0)	7226 (10.6)
2014	3034 (12.4)	1216 (15.6)	4250 (13.2)	2612 (7.3)	6862 (10.1)
**Tumour stage, N (%)**					
T1	18,029 (73.8)	3323 (42.6)	21,352 (66.3)	14,257 (39.6)	35,609 (52.2)
T2	5253 (21.5)	2558 (32.8)	7811 (24.2)	9621 (26.7)	17,432 (25.5)
T3	430 (1.8)	355 (4.6)	785 (2.4)	1581 (4.4)	2366 (3.5)
T4	113 (0.5)	146 (1.9)	259 (0.8)	1666 (4.6)	1925 (2.8)
NA	593 (2.4)	1413 (18.1)	2006 (6.2)	8892 (24.7)	10,898 (16.0)
**Outcome, N (%)**^**a**^					
Death from breast cancer (ICD-10: C50)	1040 (4.3)	883 (11.3)	1923 (6.0)	6894 (19.1)	8817 (12.9)
Death from all causes other than breast cancer (ICD-10: C50)	1457 (6.0)	391 (5.0)	1848 (5.7)	3419 (9.5)	5272 (7.7)
Death from causes other than breast cancer (ICD-10: C50) and cardiovascular diseases (ICD-10: I00-I99)	1142 (4.7)	319 (4.1)	1461 (4.6)	2677 (7.4)	4138 (6.1)
Alive	21,921 (89.8)	6521 (83.7)	28,442 (88.3)	25,699 (71.4)	54,141 (79.4)
**Follow-up, years**					
Median, IQR	7.92 (3.90)	7.18 (3.25)	7.75 (3.78)	9.78 (4.28)	8.59 (4.33)
Mean^b^, SE	7.96 (0.02)	7.29 (0.02)	7.81 (0.01)	9.28 (0.02)	8.52 (0.01)

*ICD-10* International Statistical Classification of Diseases and Related Health Problems, 10th revision, *IQR* Interquartile range, *NA* Tumours without staging information, *BC* Breast cancer, *SE* Standard error

^a^Due to rounding, the individual percentages do not always add up to the percentage of the summarised figures

^b^[28, 29]

**Fig. 2:**
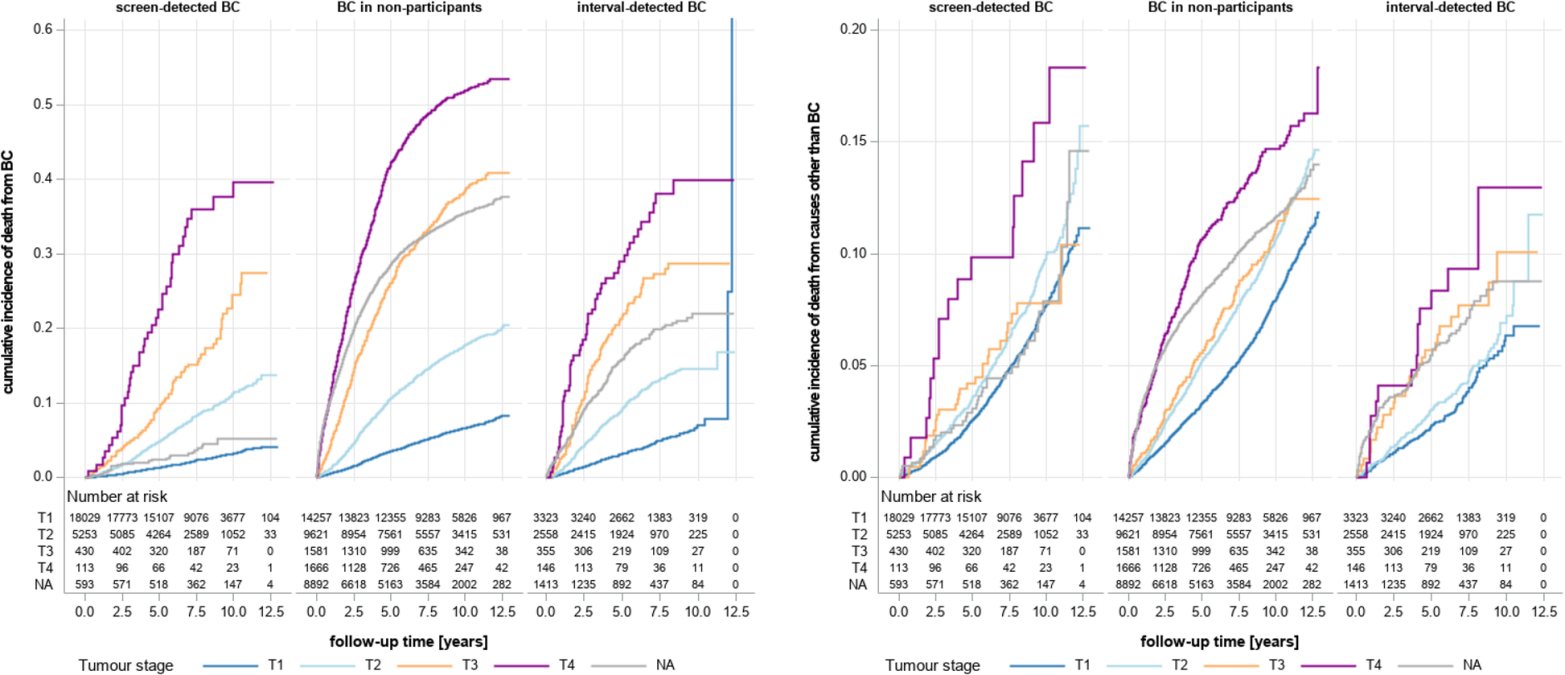
Left panel: stage-specific survival (death from breast cancer) of women with screen-detected breast cancer, non-participating women and women with interval-detected breast cancers. Right panel: stage-specific survival (death from all causes other than breast cancer) in women with screen-detected breast cancer, non-participating women and women with interval-detected breast cancers. *BC* Breast cancer, *NA* Tumours without staging information

Stage-specific survival differed across women with different detection modes. Women with screen-detected breast cancer showed a considerable survival benefit for all stages compared to non-participants with breast cancer (Fig. 2, left panel). The stage-specific comparisons between participants with interval cancer and non-participants showed smaller, but (except for T1) still obvious differences (Fig. 2, left panel). The corresponding curves of stage-specific survival generally showed an increasing divergence over time, until reaching a plateau. The higher the stage of breast cancer, the larger the difference and the earlier the plateau was reached (Fig. 2, left panel). The comparison of stage-specific survival curves for death from all causes other than breast cancer showed reduced survival for MSP non-participants compared to MSP participants with screen-detected or interval breast cancer. These differences were visible from the beginning of the observation period and stayed roughly constant over time (Fig. 2, right panel).

Tables 2, 3 and 4 show the results of the Cox models. As hazard ratios were almost identical in unadjusted models and models adjusted for age and year of diagnosis, we further report the results of the adjusted models only. Table 2 shows the comparison of breast cancer-specific survival and survival from death from all causes other than breast cancer across participants and non-participants with breast cancer. Hazards of death were considerably lower in MSP participants compared to non-participants with an HR of 0.30 (95% CI: 0.28–0.32). After adjustment for the tumour stage, this difference attenuated to an HR of 0.52 (95% CI: 0.50–0.55). For the endpoint “death from all causes other than breast cancer”, we observed a further reduction in the survival benefit in MSP participants (HR = 0.62 (95% CI: 0.58–0.66)).

**Table 2:** Effect of MSP participation status (participants versus non-participants with incident breast cancer) on death from breast cancer and all causes other than breast cancer based on *n* = 68,230 women aged 50–69 years

	**Death from breast cancer**	**Death from all causes other than breast cancer**
	**HR (95% CI)**	**HR (95% CI)**
**Models without adjustment**		
MSP non-participants	1	1
MSP participants	0.31 (0.29–0.32)	0.65 (0.62–0.69)
**Models adjusted for age at diagnosis and year of diagnosis**		
MSP non-participants	1	1
MSP participants	0.30 (0.28–0.32)	0.62 (0.58–0.66)
**Models adjusted for age at diagnosis, year of diagnosis and tumour stage**		
MSP non-participants	1	
MSP participants	0.52 (0.50–0.55)	

*MSP* Mammography screening programme, *HR* Hazard ratio, *CI* Confidence interval

**Table 3:** Effect of MSP participation status (participants versus non-participants (reference) with incident breast cancer) on breast cancer death based on *n* = 68,230 women aged 50–69 years, stratified by tumour stage

	**Death from breast cancer**
	**HR**_**participants**_** (95% CI)**
**Models without adjustment**	
T1 (*n* = 35,609)	0.53 (0.48–0.59)
T2 (*n* = 17,432)	0.65 (0.59–0.71)
T3 (*n* = 2366)	0.58 (0.49–0.69)
T4 (*n* = 1925)	0.61 (0.49–0.76)
NA (*n* = 10,898)	0.38 (0.34–0.43)
**Models adjusted for age at diagnosis and year of diagnosis**	
T1 (*n* = 35,609)	0.54 (0.48–0.60)
T2 (*n* = 17,432)	0.65 (0.59–0.71)
T3 (*n* = 2366)	0.58 (0.49–0.70)
T4 (*n* = 1925)	0.59 (0.48–0.74)
NA (*n* = 10,898)	0.38 (0.34–0.43)

*MSP* Mammography screening programme, *HR* Hazard ratio, *CI* Confidence interval

**Table 4:** Effect of detection mode on death from breast cancer and all causes other than breast cancer after incident breast cancer diagnosis based on *n* = 68,230 women aged 50–69 years

	**Death from breast cancer**	**Death from all causes other than breast cancer**
	**HR (95% CI)**	**HR (95% CI)**
**Models without adjustment**		
MSP non-participants	1	1
MSP participants with interval-detected BC	0.63 (0.59–0.68)	0.65 (0.58–0.72)
MSP participants with screen-detected BC	0.22 (0.20–0.23)	0.65 (0.62–0.70)
**Models adjusted for age at diagnosis and year of diagnosis**		
MSP non-participants	1	1
MSP participants with interval-detected BC	0.62 (0.58–0.66)	0.63 (0.56–0.70)
MSP participants with screen-detected BC	0.21 (0.20–0.22)	0.62 (0.58–0.66)
**Models adjusted for age at diagnosis, year of diagnosis and tumour stage**		
MSP non-participants	1	
MSP participants with interval-detected BC	0.70 (0.65–0.76)	
MSP participants with screen-detected BC	0.42 (0.39–0.45)	

*MSP* Mammography screening programme, *BC* Breast cancer, *HR* Hazard ratio, *CI* Confidence interval

Table 3 shows the results of the same Cox models stratified for the tumour stage. Across all tumour stages, survival is considerably better in MSP participants compared to non-participants.

Table 4 shows the results of the Cox models comparing breast cancer-specific and overall survival (i.e., survival from death from all causes other than breast cancer) across the three detection modes. Compared to non-participants, hazards of breast cancer death were considerably lower in participants with interval cancers (HR = 0.62, 95% CI 0.58–0.66) and even more in participants with screen-detected cancers (HR = 0.21, 95% CI: 0.20–0.22). These hazard ratios only partially attenuated after adjustment for tumour stage (HR = 0.70, 95% CI: 0.65–0.76 for interval cancers and HR = 0.42, 95% CI: 0.39–0.45 for screen-detected cancers). For the endpoint “death from all causes other than breast cancer” we also observed better survival in participants with interval cancers (HR = 0.63, 95% CI: 0.56–0.70) and screen-detected cancers (HR = 0.62, 95% CI: 0.58–0.66) compared to non-participants. This last comparison represents the isolated effect of confounding which can be used for correction of estimates generated in other models from similar data sources when there is no knowledge on individual confounders.

### Sensitivity analysis

When applying the endpoint “death from causes other than breast cancer (ICD-10: C50) and cardiovascular disease (ICD-10: I00-I99)” instead of “death from all causes other than breast cancer”, results remained virtually unchanged (Additional file 2: Tables S1 and S2).

## Discussion

In this analysis of cancer registry data, we disentangled the different contributing factors of the observable survival benefit of women with screen-detected breast cancer and we successfully estimated the relative contribution of confounding (or self-selection). Our data confirm that participants with screen-detected breast cancer have the largest proportion of T1 stage tumours with constantly over 70% in 2006–2014, while the higher stages, especially T3 and T4, were more frequently diagnosed in participants with interval cancers and in MSP non-participants (Additional file 1: Fig. S1). Compared with the stage distribution (T1: 47.4%) before the introduction of the MSP (2002–2004) [30], the proportion of T1 stage tumours in screen-detected participants is considerably higher, reflecting the expected MSP-related stage shift in screen-detected breast cancer.

The visual comparison of survival curves indicates the presence of several biases, namely length time bias, lead time bias and confounding (self-selection or healthy screenee bias). Reduced cancer-specific and, to a smaller degree, overall survival in MSP non-participants compared to MSP participants with screen-detected as well as interval-detected breast cancer hints towards self-selection bias in the sense of prognosis-relevant confounding in MSP participants (Fig. 2). The initial increase in divergence of corresponding survival curves and the later plateau are indicative of lead time bias, i.e., a spuriously longer survival time for women with breast cancer detected in the MSP (Fig. 2, left panel). In the comparison of the survival rates of participants with screen-detected tumours and interval-detected tumours (Fig. 2, left panel), the latter group shows a slightly reduced survival, even though both groups show a strong convergence in later years. This might be indicative of length time bias with interval breast cancer being more malignant than screen-detected tumours.

In the comparison of MSP participants and MSP non-participants (Tables 2 and 3, Fig. 2) the adjustment for tumour stage and thus elimination of stage shift leads to a reduction of the survival benefit, but MSP participants continue to have half the risk of breast-cancer-associated death compared to non-participants. As assumed by our hypothesis this probably reflects the combined effect of lead time bias, length time bias and confounding. This is supported by the results of the stratified analyses, that could not be affected by stage shift and that show the same 50% increase in survival across the different stages. Our findings are compatible with international studies (e.g., [14]), that also attribute 50–60% of breast cancer survival benefit to stage shift. For the endpoint “death from all causes other than breast cancer”, which is by design devoid of length time bias, we observe a somewhat smaller, but still considerable survival advantage of about 30% in MSP participants that must be due to confounding and a sort of “lead time” bias, that is due to the earlier start of the observation period in MSP participants with screen-detected breast cancers.

**Fig. 3:**
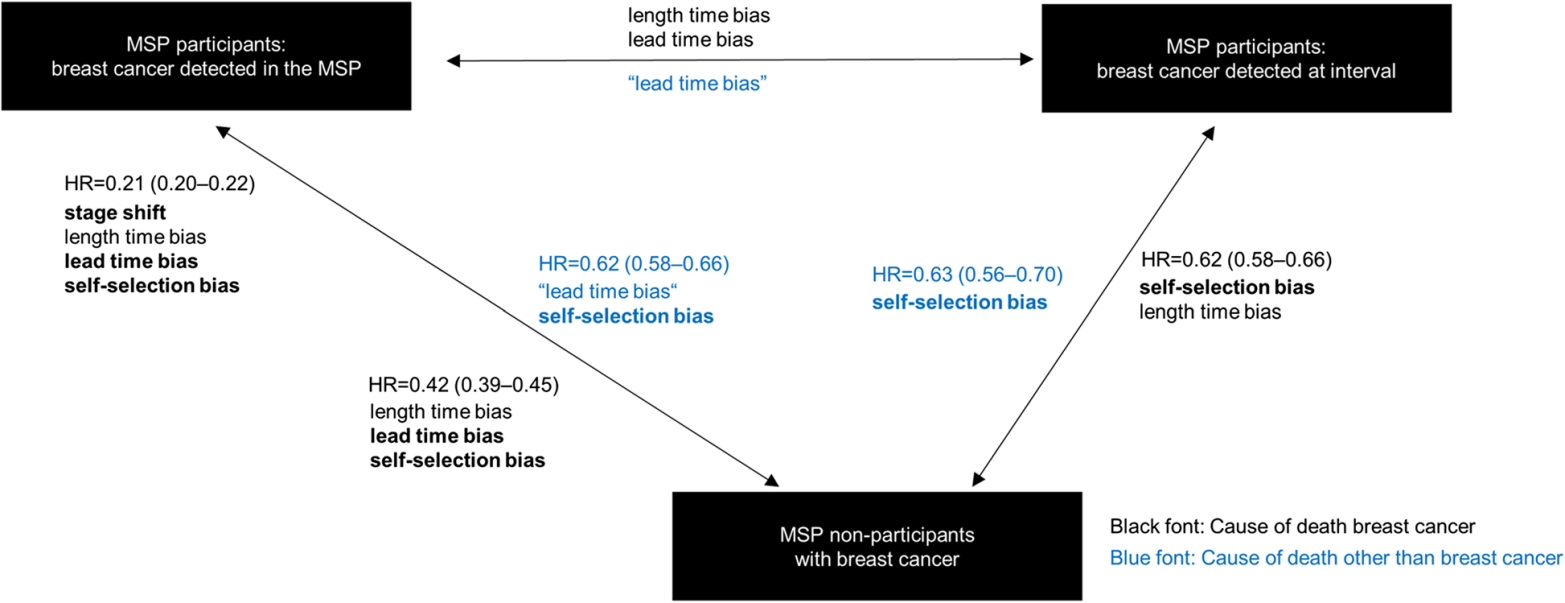
Study hypothesis combined with study findings. *MSP* Mammography screening programme, *HR* Hazard ratio with 95% Confidence interval

The effect of the different biases can be further separated when looking at different detection modes (Table 4, Fig. 3). The comparison of survival between non-participants and participants with screen-detected breast cancer is influenced by stage shift, lead time and length time bias as well as by confounding. This is reflected by a very low hazard ratio of about 0.22 (95% CI: 0.20–0.23) corresponding to a risk reduction of about 80%. This massive survival benefit becomes smaller when adjusting for the effect of stage shift, resulting in a hazard ratio of 0.42 (95% CI: 0.39–0.45). This alleged 60% risk reduction in women with screen-detected tumours compared to non-participants reflects bias. If we now look at the comparison of non-participants with participants with interval cancer instead of screen-detected cancer in the models adjusted for age and year of diagnosis, the survival advantage becomes even smaller (HR = 0.62, 95% CI: 0.58–0.66) due to the elimination of lead time bias. According to our hypothesis, this 40% risk reduction is now devoid of stage shift and lead time, but potentially affected by length time bias and confounding. To isolate the effect of confounding we now consider the endpoint “death from all causes other than breast cancer” for the same comparison (MSP participants with interval breast cancer and MSP non-participants), which is practically devoid of length time bias. The observed hazard ratio of 0.63 (95% CI: 0.56–0.70) reflects only the confounding effect, which accounts for a (spurious) risk reduction of 37% in MSP participants. As proposed, the effect of length time bias is rather small (difference between 0.62 and 0.63), as both, tumours detected in the interval and tumours in non-participants, probably include slow- as well as fast-growing tumours.

### Assumptions underlying the hypothesis

We hypothesise that we are able to isolate the proportion of observed survival advantage that is due to confounding (self-selection bias). Several assumptions are necessary for this hypothesis and the estimation of an isolated effect of confounding. First, we assume that women with interval cancers are a random subgroup of all MSP participants with respect to potential confounders. Hence, we assume that women with screen-detected breast cancer and those with cancer detected in the interval do not differ with respect to prognostically relevant confounders, but only with respect to tumour characteristics that made the tumour more or less likely to be symptomatic and affect the probability of survival [15, 31, 32]. This assumption is supported by a recent publication by Kou et al. [33] who found that, in addition to various tumour characteristics, younger age, breast self-examination, healthy weight, and use of hormone replacement therapy prior to diagnosis were the only factors that significantly differed between women with screen-detected cancers and those with interval cancers [33]. These factors may influence the likelihood of being diagnosed and may indicate different prognostically relevant tumour characteristics. However, we hypothesise that these factors are not predictors of treatment adherence and do not reflect prognostically relevant comorbidities.

Second, we assumed that interval cancers and breast cancers in non-participants are detected because of symptoms. In line with this hypothesis are again findings from Houssami and Hunter [32], who not only showed that interval tumours have poorer prognostic characteristics and survival than MSP-detected tumours, but also, that these characteristics and the associated prognosis are similar to tumours detected in MSP non-participants. It is, however, known that opportunistic screening in Germany is common in non-participants and to a lesser extent also present in MSP participants [34]. This may weaken our assumption of symptom-related diagnoses as some of the interval tumours would indeed be diagnosed through a different form of screening, e.g., by high-resolution ultrasound. This would tend to lead to an underestimation of the screening effect, but it should not affect the estimate of confounding.

Third, we assume that death from all other causes than breast cancer is affected by the same confounders or the same magnitude of confounding, respectively, as death from breast cancer. This assumption is reasonable, as most non-tumour-associated prognostic factors, such as comorbidities, health awareness and health-seeking behaviour, affect both death from breast cancer and death from all other causes, but cannot be confirmed in the absence of individual confounder information.

Taking our hypothesis one step further, we assume that the extent of confounding/self-selection bias estimated in our survival analyses corresponds to the extent of self-selection bias in comparisons of breast cancer mortality between screening participants and non-participants. Thus, the derived estimate could be used as a correction factor to adjust for confounding in corresponding mortality analyses. The key assumption necessary for this hypothesis is that women taking part in the screening programme react similarly to symptoms caused by interval tumours as women not taking part in the screening programme react to breast cancer-related symptoms.

### Comparison with other studies

Regarding the effect of confounding, the estimate of 37% in our study is comparable with other studies looking at differences in breast cancer survival or mortality due to screening. Dawson et al. [14] compared survival between screening participants and non-participants with breast cancer. They found, that after consideration of stage shift and length time bias, which explained 50–60% and 3–10% of the survival benefit in screening participants, respectively, at least 30% of the survival difference remains unexplained. Moreover, Duffy et al. [10] calculated a correction factor, that adjusts for self-selection in mammography screening participation when comparing mortality rates in absence of individual confounding information. This factor equals to 1.36, which represents roughly a 30% reduction (1 − (1/1.36) = 0.27). Many studies (e.g., [35]) use this correction factor to correct for self-selection bias in comparisons of mortality rates between screening participants and non-participants. Some other studies have calculated their own correction factor. While Paap et al. [36] calculated a much lower correction factor of 1.08 (corresponding to risk reduction of about 7%), Hense et al. [37] calculated a higher correction factor of 1.70, corresponding to a risk reduction of about 40%. Since the magnitude of the factors is comparable to the estimator calculated here (except for the factor derived by Paap et al. [36]), this would be a first indication that the extension of assumptions and thus the possible transferability of estimators from survival to mortality comparisons might be reasonable.

Moreover, the methodological approach derived in this study will be used in the evaluation of the German MSP which is currently in its final phase [38]. The evaluation concept follows a triangulation approach based on different data sources and complementary analysis concepts.

### Strengths and limitations

In Germany, there is no central cancer registry where information on the mode of cancer detection is available. Therefore, our study used long-term data from the certified and largest German cancer registry, which covers a population of more than 18 million people. Although the most recent complete dataset was used for the analyses in this study, only data up to 2018 were included. Data from cancer registries are typically not immediately available but take some time to be completed. This is particularly true for data containing information on causes of death, as these are actively linked to the official cause of death statistics, which are also available with a delay. However, as the aim of our study is to develop and illustrate a methodological approach rather than to evaluate cancer survival data, we do not consider this a limitation of our analysis.

We applied appropriate statistical methodology as well as an innovative causal framework to disentangle the different causes of observed survival benefits in screening-detected breast cancer. However, defining stage shift as the (only) intended effect of an MSP might be a strong assumption. First, there might be finer shifts in tumour sizes, that happen within stages, but nevertheless affect treatment and survival [16]. Second, besides stage shift and the described forms of bias, survival differences between MSP participants and non-participants might also arise from differences in the care pathways that follow the diagnosis of breast cancer, which would be included here in the estimated confounding effect of 37%. There is indeed evidence that screening programmes affect survival beyond the effect of early detection. Andreano et al. [39] showed that the survival probability of women with non-metastatic breast cancer is 30% higher if a guideline-based therapy is followed. Moreover, the German WiZen study showed that treatment in certified oncology centres improved the survival of cancer patients [40]. In contrast, Lundqvist et al. [41] found that survival is only marginally affected by tumour characteristics, treatment factors, comorbidity and lifestyle factors, and that the largest effect is due to confounding.

Another limitation of our study is the high proportion of missing values for tumour stage in the dataset, especially for non-screen-detected breast cancers, i.e., women with interval cancers or non-participants. Staging information for MSP participants with screen-detected cancers is obtained from the screening units, which are required by law to report tumour stage (mainly pTNM). For non-screen-detected breast cancers, pTNM and cTNM are reported by the treating hospitals, where completeness of information is not mandatory. Although we believe that the under-reporting of staging information is related to structural deficiencies of the hospitals and not to the prognostic factors of the tumours, information bias cannot be ruled out at this stage. Therefore, if for some reason missingness was also related to prognostic factors, e.g., in the way that lower or higher stage tumours were more or less likely to be underreported, the groups with non-screen-detected cancers, where most missing data occur, would on average show a systematic bias towards better or worse cancer survival, depending on the type of misclassification. However, this would not affect the analyses adjusted for or stratified by tumour stage. It would also not affect the analyses with the endpoint “death from all causes other than breast cancer”, because we would expect differences in outcome only for death from breast cancer. In summary, although there may be information bias due to the large number of missed cases of non-screen-detected breast cancer, we would not expect this to change the results of our main analysis, which is the survival comparison of women with interval cancers with non-participating women for "death from all causes other than breast cancer”.

In addition to missing information on the tumour stage, there may be differential misclassification of the tumour stage due to differences in the quality of staging examinations or documentation among the three detection mode groups. This could bias stage-specific survival comparisons. However, comparisons of average breast cancer survival (across all tumour stages) should not be affected by this bias. Similarly, the main comparison of survival between women with interval cancers and non-participants in terms of “death from all causes other than breast cancer” would not be affected by a differential misclassification of staging information.

There was also no systematic information on N and M stages in our dataset, which could have helped in providing more accurate stage-specific survival estimates. However, again this did not affect our main analysis with the endpoint “death from all causes other than breast cancer”, as stage information was not used in this analysis.

Another potential source of information bias is the classification of the outcome. The study relies on official causes of death data, which, in Germany, are only of average quality according to the criteria of the WHO [25, 26]. Thus, we cannot rule out any misclassification, particularly regarding the outcome “death from all causes other than breast cancer”, which might also include breast cancer-associated deaths. However, we do not assume this misclassification to be differential across MSP participation status or detection mode [23, 42–44]. We nevertheless carried out a sensitivity analysis with the endpoint “death from causes other than breast cancer (ICD-10: C50) and cardiovascular disease (ICD-10: I00-I99)”, which yielded essentially the same results.

We also acknowledge that the follow-up period of our study is relatively short, especially with regard to survival of lower-stage tumours. However, this does not affect the methodological approach to isolate confounding that we present. What might be affected by varying follow-up times is the magnitude of the estimated confounding, in the case that there is an interaction between tumour stage and the extent of self-selection, i.e., if the magnitude of confounding was different for different tumour stages. This could be the case, for example, if potential differences in therapy adherence between MSP participants and non-participants were greater or smaller depending on the tumour stage. If such an interaction would be present, the absolute amount of confounding would vary with varying follow-up periods. It could, however, be argued that using this approach in a given population over a given time period is an optimal measure of the extent of confounding that is actually present in that population and time period (Additional file 3: Table S1).

## Conclusions

We show here that confounding is an essential part of the observed survival benefit in women participating in MSP and should therefore be considered in future survival comparisons alongside corrections for lead and length time bias. Moreover, we show, that the extent of confounding can be estimated from a rather basic comparison of survival after breast cancer across different detection modes and that it is comparable to previously described estimates of self-selection bias in breast cancer mortality studies. It could therefore be used as a correction factor that can be easily estimated without information on the confounding factors themselves and based only on survival data from cancer registries.

### Supplementary Information

**Additional file 1: Fig. S1.** Distribution of tumour stages across the years 2006-2014 stratified by detection mode.

**Additional file 2: Table S1.** Sensitivity analysis: Effect of MSP participation status on “Death from causes other than breast cancer (ICD-10: C50) and cardiovascular diseases (ICD-10: I00-I99)”. **Table S2.** Sensitivity analysis: Effect of detection mode on “Death from causes other than breast cancer (ICD-10: C50) and cardiovascular diseases (ICD-10: I00-I99)”.

**Additional file 3: Table S3.** Number of women alive and who died of breast cancer until end of 2018, stratified by detection mode and tumour stage.

## Data Availability

The data on which this article is based cannot be made publicly available, because they are only available in the database of the Cancer Registry of North Rhine-Westphalia. Access to these data can be granted on request for health reporting and for research projects according to §§ 23 and 24 of the Cancer Registry Act of North Rhine-Westphalia. Requests for access should be addressed to the Cancer Registry of North Rhine-Westphalia, Germany.

## Abbreviations

BC: Breast cancer
CI: Confidence interval
HR: Hazard ratio
ICD-10: International Statistical Classification of Diseases and Related Health Problems, 10th revision
IQR: Interquartile range
LKR NRW: Cancer Registry of North Rhine-Westphalia
MSP: Mammography screening programme
NA: Not available (Tumours without staging information)
NRW: North Rhine-Westphalia
SE: Standard error
