# Supplementary material for: Isolating the effect of confounding from the observed survival benefit of screening participants — a methodological approach illustrated by data from the German mammography screening programme
Source: BMC Med. 2024 Jan 30;22:43. doi: 10.1186/s12916-024-03258-6 (PMC10826012; doi:10.1186/s12916-024-03258-6)
Supplement: Supplementary file 1 — Additional file 1: Fig. S1. Distribution of tumour stages across the years 2006-2014 stratified by detection mode. [file 12916_2024_3258_MOESM1_ESM.docx]

**Additional File 1** to „Isolating the effect of confounding from the observed survival benefit of screening participants – a methodological approach illustrated by data from the German mammography screening programme”

**Buschmann, Laura^1#^[
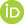
](https://orcid.org/0000-0002-8389-1253); Wellmann, Ina^2#^[
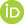
](https://orcid.org/0009-0001-1831-2542); Bonberg, Nadine^1^; Wellmann, Jürgen^1^[
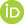
](https://orcid.org/0000-0003-3635-8584); Hense, Hans-Werner^1^[
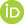
](https://orcid.org/0000-0002-7381-1547); Karch, André^1#^[
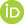
](https://orcid.org/0000-0003-3014-8543)** and **Minnerup, Heike^1#^[
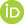
](https://orcid.org/0000-0002-9706-7599)**

^1^ Institute of Epidemiology and Social Medicine, University of Münster, Germany

^2^ State Cancer Registry North Rhine-Westphalia gGmbH, Bochum, Germany

^#^ contributed equally as first/senior authors

**Corresponding author**

Laura Buschmann

Institute of Epidemiology and Social Medicine, University of Münster, Germany

Albert-Schweitzer-Campus 1, 48149 Münster,

laura.buschmann@ukmuenster.de

[**Fig. S1:** Distribution of tumour stages across the years 2006-2014 stratified by detection mode, based on n=57,332 women aged 50-69 years with an incident breast cancer diagnosis in the years 2006-2014 I](#_Toc149512424)

**Supplementary material**

# **Fig. S1:** Distribution of tumour stages across the years 2006-2014 stratified by detection mode, based on n=57,332 women aged 50-69 years with an incident breast cancer diagnosis in the years 2006-2014

MSP: mammography screening programme

BC: breast cancer
