# Supplementary material for: Isolating the effect of confounding from the observed survival benefit of screening participants — a methodological approach illustrated by data from the German mammography screening programme
Source: BMC Med. 2024 Jan 30;22:43. doi: 10.1186/s12916-024-03258-6 (PMC10826012; doi:10.1186/s12916-024-03258-6)
Supplement: Supplementary file 2 — Additional file 2: Table S1. Sensitivity analysis: Effect of MSP participation status on “Death from causes other than breast cancer (ICD-10: C50) and cardiovascular diseases (ICD-10: I00-I99)”. Table S2. Sensitivity analysis: Effect of detection mode on “Death from causes other than breast cancer (ICD-10: C50) and cardiovascular diseases (ICD-10: I00-I99)”. [file 12916_2024_3258_MOESM2_ESM.docx]

**Additional File 2** to „Isolating the effect of confounding from the observed survival benefit of screening participants – a methodological approach illustrated by data from the German mammography screening programme”

**Buschmann, Laura^1#^[
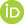
](https://orcid.org/0000-0002-8389-1253); Wellmann, Ina^2#^[
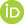
](https://orcid.org/0009-0001-1831-2542); Bonberg, Nadine^1^; Wellmann, Jürgen^1^[
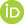
](https://orcid.org/0000-0003-3635-8584); Hense, Hans-Werner^1^[
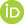
](https://orcid.org/0000-0002-7381-1547); Karch, André^1#^[
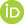
](https://orcid.org/0000-0003-3014-8543)** and **Minnerup, Heike^1#^[
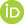
](https://orcid.org/0000-0002-9706-7599)**

^1^ Institute of Epidemiology and Social Medicine, University of Münster, Germany

^2^ State Cancer Registry North Rhine-Westphalia gGmbH, Bochum, Germany

^#^ contributed equally as first/senior authors

**Corresponding author**

Laura Buschmann

Institute of Epidemiology and Social Medicine, University of Münster, Germany

Albert-Schweitzer-Campus 1, 48149 Münster,

[laura.buschmann@ukmuenster.de](mailto:laura.buschmann@ukmuenster.de)

[**Table S1:** Effect of MSP participation status (participants versus non-participants) on “death from causes other than breast cancer (ICD-10: C50) and cardiovascular diseases (ICD-10: I00-I99)” after incident breast cancer diagnosis based on n= 68,230 women aged 50-69 years I](#_Toc155781988)

[**Table S2:** Effect of detection mode on “death from causes other than breast cancer (ICD-10: C50) and cardiovascular diseases (ICD-10: I00-I99)” after incident breast cancer diagnosis based on n= 68.230 women aged 50-69 years I](#_Toc155781989)

**Sensitivity Analyses:**

# **Table S1:** Effect of MSP participation status (participants versus non-participants) on “death from causes other than breast cancer (ICD-10: C50) and cardiovascular diseases (ICD-10: I00-I99)” after incident breast cancer diagnosis based on n= 68,230 women aged 50-69 years

|  | **Death from causes other than breast cancer and**  **cardiovascular diseases** |
| --- | --- |
|  | **HR (95 % CI)** |
| **Models without adjustment** |  |
| MSP non-participants | 1 |
| MSP participants | 0.66 (0.62-0.70) |
| **Models adjusted for age and year of diagnosis** | |
| MSP non-participants | 1 |
| MSP participants | 0.62 (0.58-0.67) |

MSP: mammography screening programme

HR: hazard ratio

CI: confidence interval

# **Table S2:** Effect of detection mode on “death from causes other than breast cancer (ICD-10: C50) and cardiovascular diseases (ICD-10: I00-I99)” after incident breast cancer diagnosis based on n= 68.230 women aged 50-69 years

|  | **Death from causes other than breast cancer and**  **cardiovascular diseases** |
| --- | --- |
|  | **HR (95 % CI)** |
| **Models without adjustment** |  |
| MSP non-participants | 1 |
| MSP participants with interval-detected BC | 0.68 (0.60-0.76) |
| MSP participants with screen-detected BC | 0.66 (0.61-0.70) |
| **Models adjusted for age and year of diagnosis** | |
| MSP non-participants | 1 |
| MSP participants with interval-detected BC | 0.65 (0.58-0.73) |
| MSP participants with screen-detected BC | 0.62 (0.57-0.66) |

MSP: mammography screening programme

HR: hazard ratio

CI: confidence interval
