# Supplementary material for: Isolating the effect of confounding from the observed survival benefit of screening participants — a methodological approach illustrated by data from the German mammography screening programme
Source: BMC Med. 2024 Jan 30;22:43. doi: 10.1186/s12916-024-03258-6 (PMC10826012; doi:10.1186/s12916-024-03258-6)
Supplement: Supplementary file 3 — Additional file 3: Table S3. Number of women alive and who died of breast cancer until end of 2018, stratified by detection mode and tumour stage. [file 12916_2024_3258_MOESM3_ESM.docx]

**Additional File 3** to „Isolating the effect of confounding from the observed survival benefit of screening participants – a methodological approach illustrated by data from the German mammography screening programme”

**Buschmann, Laura^1#^[
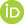
](https://orcid.org/0000-0002-8389-1253); Wellmann, Ina^2#^[
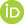
](https://orcid.org/0009-0001-1831-2542); Bonberg, Nadine^1^; Wellmann, Jürgen^1^[
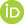
](https://orcid.org/0000-0003-3635-8584); Hense, Hans-Werner^1^[
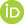
](https://orcid.org/0000-0002-7381-1547); Karch, André^1#^[
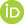
](https://orcid.org/0000-0003-3014-8543)** and **Minnerup, Heike^1#^[
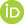
](https://orcid.org/0000-0002-9706-7599)**

^1^ Institute of Epidemiology and Social Medicine, University of Münster, Germany

^2^ State Cancer Registry North Rhine-Westphalia gGmbH, Bochum, Germany

^#^ contributed equally as first/senior authors

**Corresponding author**

Laura Buschmann

Institute of Epidemiology and Social Medicine, University of Münster, Germany

Albert-Schweitzer-Campus 1, 48149 Münster,

[laura.buschmann@ukmuenster.de](mailto:laura.buschmann@ukmuenster.de)

[**Table S1:** Number of women alive and who died of breast cancer until end of 2018, stratified by detection mode and tumour stage based on n=62,958 women aged 50-69 years, after incident breast cancer diagnosis in the years 2006-2014 I](#_Toc155781849)

**Supplementary material**

# **Table S1:** Number of women alive and who died of breast cancer until end of 2018, stratified by detection mode and tumour stage based on n=62,958 women aged 50-69 years, after incident breast cancer diagnosis in the years 2006-2014

|  | **Participants** | | | | **Non-Participants** | | **Total** |
| --- | --- | --- | --- | --- | --- | --- | --- |
|  | **Screen-detected BC** | | **Interval-detected BC** | |  | |  |
|  | **Alive** | **Death from BC** | **Alive** | **Death from BC** | **Alive** | **Death from BC** |  |
| **Tumour stage N (%)** |  |  |  |  |  |  |  |
| T1 | 16,599 (97.4) | 440  (2.6) | 3,032  (95.0) | 161  (5.0) | 12,306  (93.9) | 883  (6.1) | 33,421 |
| T2 | 4,409  (90.5) | 461  (9.5) | 2,124  (87.2) | 313  (12.8) | 7,043  (81.5) | 1,596  (18.5) | 15,946 |
| T3 | 326  (81.3) | 75  (18.7) | 236  (72.0) | 92  (28.0) | 863  (60.5) | 563  (39.5) | 2,155 |
| T4 | 58  (59.8) | 39  (40.2) | 79  (60.3) | 52  (39.7) | 602  (42.0) | 830  (58.0) | 1,660 |
| NA | 529  (95.5) | 25  (4.5) | 1,050  (79.8) | 265  (20.2) | 4,885  (61.8) | 3,022  (38.2) | 9,776 |
| Total | 21,921 | 1,040 | 6,521 | 883 | 25,699 | 6,894 | 62,958 |

BC: breast cancer

NA: tumours without staging information
